# Mirror neuron therapy for hemispatial neglect patients

**DOI:** 10.1038/srep08664

**Published:** 2015-03-02

**Authors:** Wei Wang, Xin Zhang, Xiangtong Ji, Qian Ye, Wenli Chen, Jun Ni, Guangyu Shen, Bing Zhang, Ti-Fei Yuan, Chunlei Shan

**Affiliations:** 1 Department of Rehabilitation Medicine, First Affiliated Hospital of Nanjing Medical University, Nanjing. 210029, China; 2 Department of Radiology, The Affiliated Drum Tower Hospital of Nanjing University Medical School, 321 Zhongshan Road, Nanjing, China; 3 The affiliated hospital of Nantong University, Nantong, China; 4 School of Psychology, Nanjing Normal University, Nanjing. 210097, China; 5 Shanghai University of Traditional Chinese Medicine, Shanghai. 201203, China

## Abstract

Mirror neuron system(MNS) based therapy has been employed to treat stroke induced movement disorders. However, its potential effects on patients with hemispatial neglect were uninvestigated. The present study set out to test the therapeutic efficiency of video watching of series of hand actions/movements (protocol A) in two patients with left hemispatial neglect, due to the right hemisphere stroke. The video containing dynamic landscape of natural scene or cities but not human/animals was used as the protocol B. The “ABA” training procedure for 3 weeks therefore allows us to internally control the individual differences. Before and after each week of training, the Chinese behavioral inattention test- Hongkong version (CBIT-HK) was implemented to evaluate the hemispatial neglect severity. Functional magnetic resonance imaging (fMRI) experiment was implemented in two health subjects to reveal the difference of brain activation between protocol A and B. The results showed that protocol A rather than protocol B significantly improved the CBIT-HK scores at first and third weeks, respectively. Protocol A induced more bilateral activations including right inferior parietal lobe (supramarginal gyrus), which belongs to MNS and is also critical region resulting to hemineglect. Conclusion: MNS activation can provide a novel therapy for hemispatial neglect patients.

The unilateral neglect (hemineglect) is also defined as unilateral spatial neglect or hemispatial neglect, which includes the loss of visual perception to objects within the visual area opposite to lesion hemisphere(commonly right lesion and left hemineglect). Hemispatial neglect is common as complications of patients following brain injuries^1^, and the incidence ranged 10%–82% for right hemisphere stroke patients^2^, mainly as 40%^3^,^4^. Right inferior parietal lobule is considered as the main brain region responsible for left hemispatial neglect^4^,^5^,^6^,^7^. The presence of hemispatial neglect leads to adverse effects on motor activities, and the life of quality in patients^8^.

Mirror neuron is one of the most important discoveries in neuropsychology field. Mirror neuron will fire both when executing the movement (e.g. hand movement) and observing the same movement^9^. Mirror neuron is therefore considered as important neural substrate for action understanding, imitation, language learning and empathy. Mirror neuron system (MNS) mainly includes inferior frontal gyrus (BA44), premotor cortex (BA6), and inferior parietal lobule (BA39,40) in the brain^9^. It has been found that mirror neuron system activation could lead to improvements in motor functions in stroke patients^10^,^11^, as well as language functions of stroke patients with aphasia (unpublished data). Given the fact that hemispatial neglect is due to the dysfunction of inferior parietal lobule (critical MNS areas), we wonder if the activation of MNS by hand action observation could result in improvements of hemispatial neglect symptoms.

## Methods

### Behavioral experiment

#### Clinical data

The study has been performed according to the guidelines of clinical medical research in Nanjing Medical University and has been approved by the ethic committee of medical research on human subjects in Nanjing Medical University. Informed written consents were obtained from both cases.

**Figure 1:**
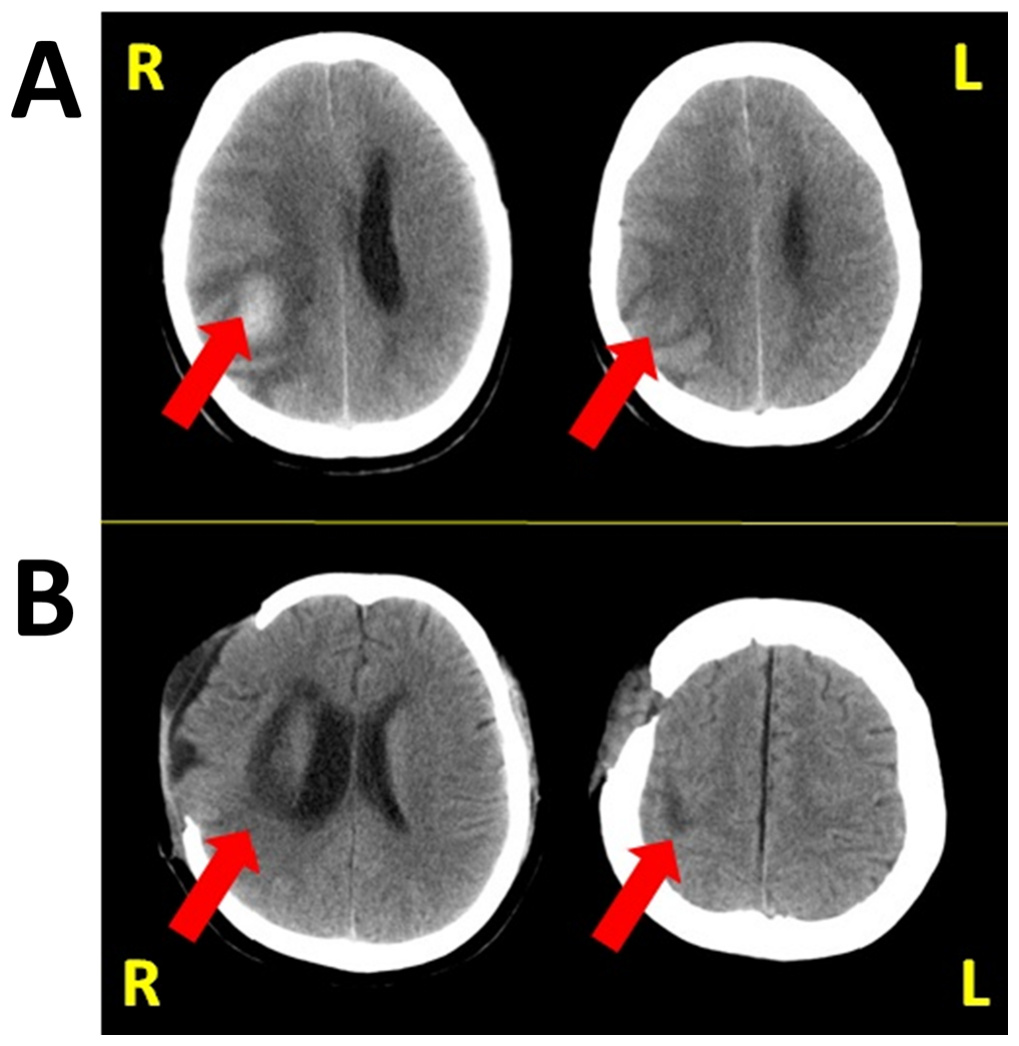
The image showing stroke sites in the two cases. (1A): case 1. (1B): case 2.

Case 1: Male, 64 years old, right handed, middle school education. The patient was admitted to the hospital on Jan 2014 due to the sudden headache, vomiting and left side body paralysis. The head CT revealed hemorrhage at the right occipital and parietal lobe (see Fig. 1A). The patient was treated to lower the intracranial pressure, nervous system nutrition, improve the micro-circulation. The patient was then diagnosed as left hemispatial neglect. On Feb 2014, the Brunnstrom stages: Left upper limb III, left hand IV, and left lower limb IV. The mini-mental state examination (MMSE) scored 29. The patient also received muscle stretch training, balance training, standing training, and other routine activities of daily rehabilitation.

Case 2: Female, 45 years old, right handed, middle school education. The patient was admitted to the hospital on Oct 2013 due to the sudden left paralysis and headache. The head CT revealed hemorrhage of the right basal ganglia and parietal lobe, and the intracranial hematoma removal plus decompressive craniectomy were performed (see Fig. 1B) (Figure 1). The patient was then diagnosed as left hemispatial neglect. On Feb 2014, the Brunnstrom stages: left upper limb III, left hand III, and the left lower limb III. The MMSE scored 25. The patient also received muscle stretch training, balance training, standing training, and other routine activities of daily rehabilitation.

#### Videos for training

Protocol A: Hand action videos include 105 videos of daily life movements, lasting for 8 seconds each with no directional preference (left-right hand balanced). The videos were played sequentially and repeated after 2 minutes. The total session of training lasted for 30 minutes.

Protocol B: The control videos include dynamic sights of mountains, rivers, forest, clouds and city sights but no human/animal. The total length of the video lasted for 30 minutes (2 minute rest in the middle, same as Protocol B).

#### Training procedure and evaluation

The two patients received three weeks training procedure in “ABA” sequence, with one protocol for a week each (6 days a week). During the training, the patients sit in the midline in front of the computer and watch the videos for 30 minutes.

The evaluation was performed single-blindly. The Chinese behavioral inattention test-Hong Kong version (CBIT-HK) was used, which included 6 conventional tests (line crossing, letter cancellation, star cancellation, figure and shape copy, line bisection and representational drawing) (146 points in total), and 9 behavioral tests (pictures scanning, telephone dialing, menu reading, article reading, telling and setting the time, coin sorting, address and sentence copying, map navigation, card sorting) (81 points in total). The total score of CBIT-HK test was 227 points, and a lower score would indicate more severe form of hemispatial neglect.

#### Behavioral Data Statistics

The data were presented as mean and SD, and analyzed by SPSS 21.0 software (Chicago, US). The correct rate was compared with chi-square test. P < 0.05 was considered as statically significant.

### fMRI experiment

#### Participants and fMRI paradigm design

2 healthy subjects (1male) (both are 24 years old, right handed) were included in fMRI experiment.

**Figure 2:**
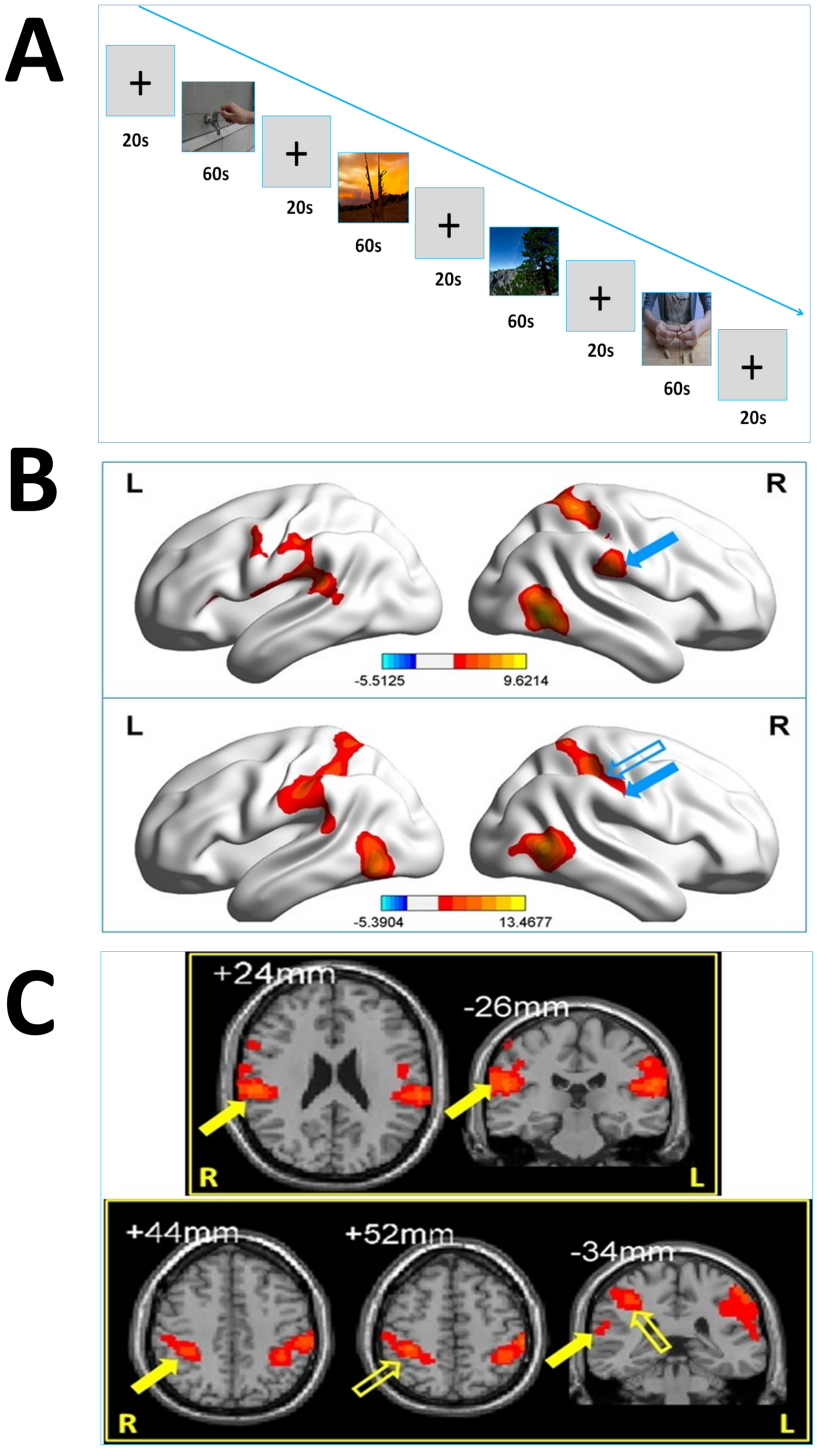
fMRI experiment. (2A): Demo of the run of video blocks (This is Run 1, i.e. Protocol ABBA. In Run2, is Protocol BAAB). The images were taken by the authors, and the hand picture is from Xiangtong Ji. (2B): illumination of distinct activations by Protocol A (stronger than Protocol B); upper is JXT and lower is ZSC. Blue solid arrow is corresponding to the Supramarginal Gyrus (BA 40) and blue hollow arrow is corresponding to the right Inferior Parietal Lobule. (2C): 2D illumination of distinct activations by Protocol A (stronger than Protocol B), upper is JXT and lower is ZSC. Yellow solid arrow is corresponding to the Supramarginal Gyrus(BA 40) and yellow hollow arrow is corresponding to the Right Inferior Parietal Lobule.

During the fMRI scanning, the two subjects were presented two runs of blocks of hand actions video and dynamic landscape of natural scene alternatively (see Fig. 2A) Each block of video last 60 s (20 video fragments, each is 3 s) and the rest block (see crosshair “+”) last 20 s. Therefore, the total time of each run is 340 s.

In order to confirm the subjects watched the videos continuously and were not out of mind, they were asked to put down a button in the response box as soon as they saw the video of playing basketball (20% in Protocol A) or flying balloon(20% in Protocol B).

#### MRI data acquisition

The studies were performed on a Philips Achieva 3.0 T TX dual Medical Systems using an 8-channel phased array coil. MR images of the entire brain were acquired using EPI with the following parameters: TR = 2000 ms, TE = 30 ms, Flip Angle = 90°, FOV = 192*192*140 mm^3^, Acquisition Matrix = 64*64, number of slices = 35, slice thickness = 4 mm and number of repetitions = 180. A 3D T1-weighted, high-resolution anatomical image set was also acquired from each subject for functional map overlay with the following parameters: TR = 9.8 ms, TE = 4.6 ms, Flip Angle = 8°, FOV = 200*200*192 mm^3^, Acquisition Matrix = 200*180, number of slices = 192, slice thickness = 1 mm.

#### fMRI processing and analysis

The fMRI images were re-aligned, co-registered and normalized to the Montreal Neurological Institute brain template using SPM8 [http://www.fil.ion.ucl.ac.uk/spm/]. The fMRI images were normalized, re-sliced into 3*3*3 mm^3^ voxels before applying a smoothing Gaussian kernel of 8*8*8 mm^3^ (full width at half maximum). Activation maps for each subject, were computed using general linear models (GLMs) and used in a first level random effects analysis to generate activation maps (P < 0.05, Alphasim correction, Number of Clusters >389).

## Results

### Behavior performance on BIT

We found that in compared to pre-test, the protocol A training at the first and third weeks significantly improved conventional test score, behavioral test score and the total CBIT-HK scores (P < 0.05), while the protocol B training did not (except for total score in case 2).

In compared to the second week training with protocol B, the protocol A training at the third week exhibited benefits in all aspects (P < 0.05) (Table 1).

### fMRI Results and Comparison of fMRI activations between Protocol A and B

Common activations induced by Protocol A and B include bilateral occipital, ventral occipitotemporal, premotor cortex and left superior parietal cortex. The difference of activation areas or stronger activations in Protocol A rather than Protocol B are showed in Fig. 2B.

The Fig. 2B and 2C indicated that for JXT(upper), Protocol A induced more activations in left ventral primary motor and sensory area (BA4, BA1–3), left inferior parietal lobule(supramarginal gyrus, BA 40), left Wernicke area(superior temporal gyrus, BA22), right superior parietal lobule (BA5), right inferior parietal lobule(supramarginal gyrus, BA 40) and right ventral occipitotemporal cortex (BA37).

The Fig. 2B and 2C also illustrated that for ZSC (lower), Protocol A induced more activations in left superior lobule (BA 5), left inferior parietal lobule (including supramarginal gyrus, BA 40), left Wernicke area(superior temporal gyrus, BA22), left ventral occipitotemporal cortex (BA37), right superior parietal lobule(BA5), right inferior parietal lobule(including upper part of supramarginal gyrus, BA 40) and right ventral occipitotemporal cortex (BA37).

The cluster volume, MNI coordinates of activations in the Right Inferior Parietal Lobule or Right Supramarginal Gyrus (BA 40) of JXT and ZSC were showed in Table 2 (Table 2).

## Discussion

In recent years, the mirror neuron system (MNS) activation has been employed in brain rehabilitation from stroke, especially for motor function and language functions^10^,^11^,^12^. However, to our knowledge, the present study is the first to explore the potential benefits of MNS therapy on hemispatial neglect. We found that the protocol A resulted in clear and better rehabilitation effects on all cases presented, in compared to the protocol B. Not only from literature^9^ but also from fMRI experiment results in this study, we can believe that hand action observation (Protocol A) could induce or induce more (than Protocol B) activations in MNS especially in inferior parietal lobule(including supramarginal gyrus BA40), which is the most critical lesion site for hemineglect^4^,^5^,^6^,^7^. This proved that MNS activation therapy could potentially treat hemispatial neglect.

The neural mechanisms underlying MNS activation therapy are unknown. It is proposed that the activation of MNS led to brain plasticity, potentially mediated by glutamatergic and neurotrophic mechanisms – which were found to be important in activity dependent brain plasticity^12^,^13^,^14^. In addition, right inferior parietal lobule belongs to the MNS, the activation of which might facilitate the functioning of this brain region, and therefore improve the relative spatial perception or attention function. Whether the MNS therapy contributes to facilitated brain reorganization is yet to be understood in future studies.

We found that one week training with protocol A was sufficient to improve the visual spatial perception in the patients. In fact, it is shown that the hemispatial neglect could be partly treated with short-period training (e.g. 2 days or 4 days prism training); on the other hand, this is in accordance with the fact that MNS activation therapy was able to improve the motor function and language function in very short period (1–2 weeks)^15^,^16^.

The present study adopted “ABA” sequence for video training. ABA is highly suitable for studies in department of rehabilitation, given the same hospitalization period for most patients; in addition, ABA is used to judge for the effects of self-recovery, tiring effect on training. Therefore the score decrease at the second week excluded the possibility of self-recovery in the patients.

The present study however requires more cases with longer-training period and longitudinal fMRI experiments for hemineglect patients in future experiments.

## Author Contributions

W.W., X.J., Q.Y., W.C., B.Z., T.Y. and C.S. designed the study; W.W., X.J., Q.Y., W.C., J.N., G.S., T.Y. and C.S. performed the study; W.W., J.N., G.S., T.Y. and C.S. analyzed the data; W.W., T.Y. and C.S. wrote the manuscript; all authors have read the approved the final version of the manuscript.

## Figures and Tables

**Table 1:** The conventional and behavioral test scores

	Conventional test		Behavioral test		CBIT-HK total score	
	Case1	Case 2	Case 1	Case 2	Case 1	Case 2
Pre-test	40	45	8	15	48	60
1 week	61^a^	75^a^	19^a^	31^a^	80^a^	106^a^^b^
2 weeks	48	61	15	23	63	84 a
3 weeks	70^a^^b^	82^a^^b^	31^a^^b^	44^a^^b^	101^a^^b^	126^a^^b^

Note: ^a^P < 0.05 in compared to pre-test;

^b^P < 0.05 in compared to 2 weeks.

**Table 2:** Activations in the Right Inferior Parietal Lobule or Right Supramarginal Gyrus (BA 40)

Subject	Brain Area	Cluster Volume(mm3)	MNI Coordinates(x, y, z)	T	Z
JXT	R Supramarginal Gyrus	4887	61, −24, 24	5.13	5.03
ZSC	R Supramarginal Gyrus	1782	36, −35, 44	4.91	4.82
	R Inferior Parietal Lobule	3078	45, −35, 54	3.94	3.89
